# Complications after plate fixation and elastic stable intramedullary nailing of dislocated midshaft clavicle fractures: a retrospective comparison

**DOI:** 10.1007/s00264-012-1615-5

**Published:** 2012-07-31

**Authors:** Frans-Jasper Wijdicks, Marijn Houwert, Marcel Dijkgraaf, Diederik de Lange, Koen Oosterhuis, Geertjan Clevers, Egbert-Jan Verleisdonk

**Affiliations:** 1 Department of Surgery, Diakonessenhuis Utrecht, Utrecht, The Netherlands; 2 Clinical Research Unit, Academic Medical Centre Amsterdam, Amsterdam, The Netherlands; 3 Department of Surgery, Ruwaard van Putten Ziekenhuis, Spijkenisse, The Netherlands

## Abstract

**Purpose:**

The incidence of operative treatment of dislocated midshaft clavicle fractures (DMCF) is rising due to unsatisfactory results after non-operative treatment. Knowledge of complications is important for selection of the surgical technique and preoperative patient counselling. The aim of this study is to compare complications after plate fixation and elastic stable intramedullary nailing (ESIN) with a titanium elastic nail (TEN) for DMCF.

**Methods:**

A retrospective analysis of our surgical database was performed. From January 2005 to January 2010, 90 patients with DMCF were treated with plate fixation or ESIN. Complications were evaluated in both treatment groups and subsequently compared.

**Results:**

Seven implant failures occurred in six patients (14 %) of the plate group and one implant failure (2.1 %) was seen in the ESIN group (*p* = 0.051). Major revision surgery was performed in five cases in the plate group (11.6 %) and in one case (2.1 %) in the ESIN group (*p* = 0.100). Three refractures (7.0 %) were observed in the plate group after removal of the implant against none in the ESIN group (*p* = 0.105). Six minor revisions (13 %) were reported in the ESIN group and none were reported in the plate group (*p* = 0.027).

**Conclusions:**

Compared to other studies we report higher rates of refracture (7.0 %), major revision surgery (11.6 %) and implant failure (14.0 %) after plate fixation. The frequency of implant failures differed almost significantly for patients treated with plate fixation compared to ESIN. Furthermore, a tendency towards refracture after implant removal and major revision surgery after plate fixation was observed.

## Introduction

Clavicle fractures occur commonly; between 2.6 and 10 % of all fractures are clavicle fractures [1]. Operative treatment for dislocated midshaft clavicle fractures (DMCF) is increasing due to reported unsatisfactory results after non-operative treatment [2–4]. Two recently published randomised trials have proven the superiority of both plate fixation and elastic stable intramedullary nailing (ESIN) over non-operative treatment for DMCF in terms of functional outcome and pain relief [5, 6].

The two most commonly used techniques for operative treatment of DMCF are plate fixation and ESIN [7]. Plate fixation results in a biomechanically stable construction allowing early mobilisation and providing for fracture compression. Long-term outcome and experience with this procedure have been well documented [8]. Complications associated with plate fixation are refracture of the clavicle after implant removal and wound infection [9, 10].

ESIN is a relatively new and technically more demanding technique [7]. If closed fracture reduction is possible, ESIN has the advantage of maintaining an intact fracture haematoma which could speed up fracture healing. If open fracture reduction is necessary, surgical incisions are in general smaller in comparison to plate fixation resulting in improved cosmetic results. In addition, smaller incisions may result in lower infection rates [6, 11]. Possible disadvantages of ESIN are medial nail protrusion and the need for implant removal requiring a second operation [11, 12].

Knowledge of possible complications is essential on the appropriate surgical technique and preoperative patient counselling. A recent Cochrane review showed that comparative studies of different techniques for operative treatment of DMCF are lacking [13]. The aim of this study is to retrospectively compare complications after plate fixation and ESIN with a titanium elastic nail (TEN) for DMCF.

## Materials and methods

A retrospective analysis of data from the surgical database at our hospital was performed. The Diakonessenhuis is a level 2 trauma centre and a regional teaching hospital. All mono-trauma patients who underwent operative treatment for a DMCF between January 2005 and July 2010 were eligible for inclusion. This inclusion period allowed for another year of follow-up postoperatively. Dislocation was defined as at least one shaft width difference in height between the fracture parts, regardless of the reduction.

The following exclusion criteria were used: (1) patients with pre-existent morbidity concerning the arm, shoulder or hand, (2) open fractures, (3) pathological fractures, (4) presence of neurovascular injury and (5) fractures older than one month or non-unions.

### Operative treatment

The operations were performed or supervised by one of the trauma surgeons (DL, KO, GJC, EV). The choice of the procedure was based on either the surgeon’s or patient's preference.

### Operative technique: plate fixation

Patients were treated according to the principles set by the AO Foundation. Patients were administered prophylactic antibiotics. A compression plate with additional interfragmentary lag screws or a bridging plate in cases of severe comminution were used for different kinds of fracture types. A transverse incision was made over the fracture site and in all cases the plate was positioned on the anterior superior surface of the clavicle. Different types of plates were used, which were provided by Synthes® b.v., Zeist, Netherlands. In the beginning of the study period reconstruction plates with the corresponding screws were used, later on small fragment locking plates with the corresponding screws became available and for the remaining time of the study these plates and screws were used.

### Operative procedure ESIN

Patients were administered prophylactic antibiotics. A small skin incision was made approximately 1 cm lateral to the sternoclavicular joint. For ESIN a Synthes® TEN was used. A TEN was inserted with a diameter varying from 2 to 3.5 mm, depending on the width of the bone. Closed reduction, initially fixed with two percutaneously pointed reduction (Weber) clamps, was performed and confirmed by fluoroscopy. If closed reduction failed, an additional small incision was made above the fracture site for direct manipulation of the main fragments. After complete introduction of the TEN into the lateral fragment, the fracture was compressed and the TEN was cut as short as possible at the medial end.

### Postoperative management

The choice of postoperative management was based on the surgeon’s preference, but patients generally received a sling while being encouraged to start early mobilisation if pain permitted.

### Complications

The complications were divided into two groups: major and minor complications.

### Major complications

The following complications were regarded as major (Table 1): non-union, symptomatic malunion, refracture after implant removal, deep infections or breakage of the implant.

**Table 1:** Major and minor complications

Major complications	Minor complications
Non-union	Superficial infection
(Symptomatic) malunion	Pain after 6 months
Implant fracture (breakout)	Temporary brachial plexus lesion
Major revision surgery	Hyperaesthesia
Deep infection	Plate irritation
Refracture after implant removal	Medial TEN protrusion
	Lateral TEN protrusion
	Minor revision surgery (shortening of TEN)

Non-union was defined as an unsuccessful healing of the bone after six months that clinically could be associated with pain and was visible on the radiograph as a gap between the fracture parts. Symptomatic malunion was defined as an incorrect anatomical position of the clavicle in comparison to the (healthy) side resulting in pain symptoms or a loss of function of the shoulder. Implant-related problems such as breakage were determined on the radiographs. Deep infection was defined as infection requiring implant removal. Refracture was defined as a fracture of the clavicle after implant removal and diagnosis was based on clinical symptoms and radiographs.

### Minor complications

The following complications were considered as minor (Table 1): (oral) antibiotics for a wound infection or cutting the protruding end of the TEN under a local anaesthetic in case of medial irritation or skin perforation. Other minor complications were deep infections not requiring implant removal or debridement, migration and telescoping (for ESIN), angulation of the implant without persistent symptoms and neurovascular problems.

Infection was defined as redness, swelling, purulent discharge, a positive wound culture and/or when prescription of antibiotics was given. Irritation (of the skin) was assessed clinically and caused by prominence of the implant material or in case of the TEN medial or lateral protrusion. Migration was defined as the medial or lateral displacement of the TEN without movement of the fracture parts also resulting in medial or lateral protrusion. Telescoping was defined as displacement of the fracture parts and the TEN. Pain was considered significant if it was still present after six months.

When treated with ESIN the TEN was always removed after four months and/or if consolidation was achieved. Plate fixation was only considered for removal after consolidation and if patients experienced irritation or nuisance caused by the implant, or by explicit request of the patient. Consolidation and appropriate time of removal were determined by the treating surgeon by examining radiographs and the clinical condition of the patient.

### Statistical analysis

Descriptives were reported as mean and standard deviation (SD) or as median and interquartile range (IQR), depending on normal or non-normal distributions of the data, respectively. An independent samples *t* test was performed to assess differences in age between groups; the Mann–Whitney U test was performed in case of total follow-up duration; the chi-square test was used in case of gender, fracture side, trauma mechanism and ≥1 irritation(s); and Fisher’s exact test was used in case of AO Classification, neurovascular injury, imminent skin perforation, ≥1 implant failure(s), major surgical revision, refractures, superficial infection and minor revision surgery. Kaplan-Meier survival analysis was performed to assess differences in time to removal of TEN or plate fixation, with non-removal considered as censored observation. The level of significance was set at *p* < 0.05. Statistical analyses were performed using SPSS software (version 17.0.0, Chicago, IL, USA).

### Approval

In accordance with the legal department of the Diakonessenhuis Utrecht and the local Ethics Commission, individual patient approval was not required due to full anonymity of the included patients and the retrospective study design.

## Results

**Fig. 1:**
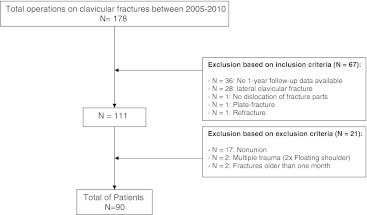
Flowchart: selection of patient group

According to the inclusion and exclusion criteria 90 patients could be included in the analysis (Fig. 1); 43 patients were treated by plate fixation and 47 patients were treated using ESIN. On average, patients in the plate group were 39.4 (SD 14.1) years of age and older (*p* = 0.049) than patients in the ESIN group, who were 33.1 (SD 15.6) years of age (Table 2). In the ESIN group closed fracture reduction was performed in seven cases (14.9 %), and open fracture reduction was performed in the remaining 40 patients (85.1 %). The median follow-up time of all patients was seven months (IQR 4–13 months). The plate group had a median follow-up time of eight months (IQR 2–13 months). A median follow-up time of six months (IQR five to 12 months) was observed in the ESIN group.

**Table 2:** Baseline characteristics, follow-up duration and time to removal for both treatment groups

		Plate group, *n* = 43	ESIN group, *n* = 47	*p* value
Age, years (mean ±SD)		39.4 ± 14.1	33.1 ± 15.6	*p* = 0.049
Gender, *n* (%)	Male	33 (77)	33 (70)	*p* = 0.48
	Female	10 (23)	14 (30)	
Fracture side, *n* (%)	Right	20 (47)	19 (40)	*p* = 0.56
	Left	23 (54)	28 (60)	
AO Classification, *n* (%)	A1	–	–	*p* = 0.11
	A2	11 (26)	22 (47)	
	A3	8 (19)	7 (15)	
	B1	1 (2)	0	
	B2	17 (4%)	14 (30)	
	B3	2 (5)	4 (9)	
	C1	1 (2)	0	
	C2	3 (7)	0	
	C3	–	–	
Trauma mechanism, *n* (%)	Traffic accident	9 (21)	12 (26)	*p* = 0.81
	Sports	18 (42)	18 (38)	
	Fall	13 (30)	12 (26)	
	Unknown^a^	3 (7)	5 (11)	
Neurovascular injury, *n* (%)		0	2 (4)	*p* = 0.23
	Unknown^a^	3 (7)	9 (19)	
Imminent skin perforation, *n* (%)		1 (2)	2 (4)	*p* = 0.61
	Unknown^a^	3 (7)	9 (19)	
Follow-up time, months (median, IQR)		8 (2–13)	6 (5–12)	*p* = 0.76
Time to removal, months (median, IQR)		11 (7–15)	5 (4–6)	*p* = < 0.001

*TEN* titanium elastic nail, *SD* standard deviation, *AO Classification* Müller AO Classification for Fractures—Long Bones, *ESIN* elastic stable intramedullary nailing

^a^Was not reported in documentation

### Major complications

#### Implant failure

**Fig. 2:**
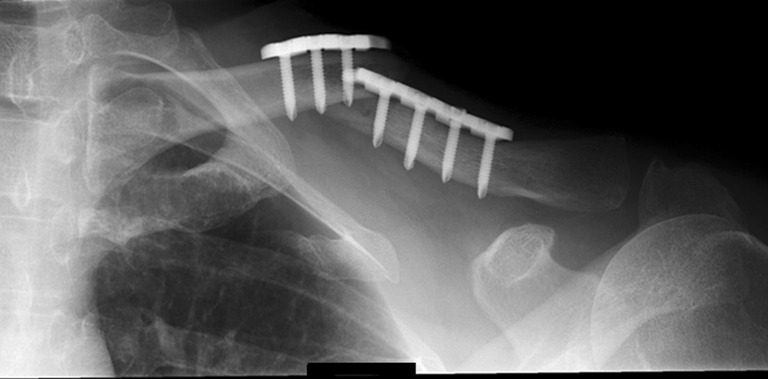
Example of a broken 3.5-mm reconstruction plate

Seven implant failures occurred in six patients (14.0 %) of the plate group. One implant failure was seen in the ESIN group (2.1 %) (*p* = 0.051, Table 3). All implant fractures occurred within three months of the primary surgical procedure (Fig. 2).

**Table 3:** Major and minor complications in both treatment groups

Major complications	Plate group, *n* = 43	ESIN group, *n* = 47	*p* value
At least 1 implant failure^a^, *n* (%)	6 (14)	1 (2)	*p* = 0.051
Major revision surgery, *n* (%)	5 (12)	1 (2)	*p* = 0.100
Refracture after implant removal, *n* (%)	3 (7)	0	*p* = 0.105
Minor complications			
Superficial infection, *n* (%)	1 (2)	4 (9)	*p* = 0.363
At least one irritation, *n* (%)	19^b^ (44)	29^b^ (62)	*p* = 0.096
Pain after 6 months, *n* (%)	4 (9)	2 (4)	
Temporary brachial plexus lesion, *n* (%)	0	2 (4)	
Hyperaesthesia, *n* (%)	3 (7)	0	
Plate irritation, *n* (%)	17 (40)		
Medial TEN protrusion, *n* (%)		23 (49)	
Lateral TEN protrusion, *n* (%)		3 (6)	
Minor revision surgery (shortening of TEN), *n* (%)	0	6 (13)	*p* = 0.027

*TEN* titanium elastic nail, *ESIN* elastic stable intramedullary nailing

^a^One patient experienced two implant fractures

^b^Some patients experienced more than one irritation problem

**Fig. 3:**
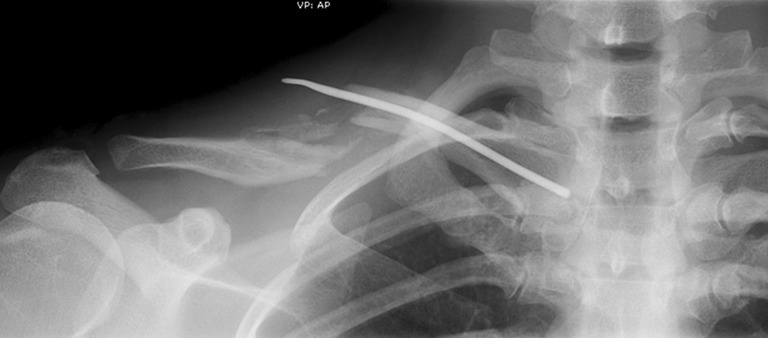
Example of implant failure of a TEN

Three of these broken plates were revised with plate fixation and supplementary cancellous bone graft. The remaining four implant failures in the plate group were treated conservatively in all cases after removal of the implant. One of these patients recovered with a slightly impaired shoulder function, and the other patients all healed uneventfully. The implant failure in the ESIN group (Fig. 3) was revised using plate fixation and supplementary bone graft and healed uneventfully.

#### Major revision surgery

Major revision surgery was performed in five cases in the plate group (11.6 %), and one major revision surgery was performed in the ESIN group (2.1 %) (*p* = 0.100, Table 3). Three revision operations were performed in the plate group as mentioned above due to implant failure (see Implant failure). One major revision operation in the plate group was the treatment of a refracture after removal of the implant (see Refractures). The last major revision operation was performed due to a complicated removal of the implant, requiring the use of a carbide drill in an additional operation. The major revision surgery in the ESIN group was due to implant failure and revised with plate fixation and spongiosa transplantation (see Implant failure).

#### Refractures

Three refractures (7.0 %) were observed in the plate group after removal of the implant against none in the ESIN group (*p* = 0.105, Table 3). All refractures occurred within 2 months after removal of the implant. Two refractures were treated conservatively and one refracture was treated with plate fixation (see Major revision surgery); all three healed uneventfully.

#### Other major complications

No other major complications such as bone healing problems or deep infections occurred.

### Minor complications

Six minor revisions (13 %) were reported in the ESIN group and none were reported in the plate group (*p* = 0.027, Table 3). The definition of minor revisions is cutting the protruding medial end of the TEN under a local anaesthetic. These procedures were performed as a result of irritation of the implant (three patients) and medial skin perforation (three patients). One of the patients with medial skin perforation was also diagnosed with superficial wound infection, which was treated with oral antibiotics and healed uneventfully.

#### Removal

In the ESIN group 45 TEN of the total 47 TEN were removed. In the plate group 27 patients (total of 43 patients) underwent removal of the implant material. The median time until removal in all 72 patients was six months (IQR 4–11). In the ESIN group the implants were removed at a median of five months (IQR 4–6). Plates were removed at a median of 11 months (IQR 7–15) (*p* < 0.001, Table 2).

## Discussion

Implant failure just fell short of being significantly more frequently observed after plate fixation (*p* = 0.051). Similarly, refracture after implant removal and major revision surgery just tended to prevail more often after plate fixation. Moreover, 80.0 % of the revision procedures were due to implant failure. Minor revision surgery on the other hand was more frequently observed after ESIN (*p* = 0.027).

Compared to other studies we report higher rates of refracture (7.0 %), major revision surgery (11.6 %) and implant failure (14.0 %) after plate fixation. Ferran et al. reported no implant failures [14]. The Canadian Orthopaedic Trauma Society reported one (1.6 %) case of early mechanical failure, Chen et al. reported 7.1 % implant failure and Liu et al. reported an implant failure rate of 8.5 % [5, 15, 16]. Our results are comparable with the results of Böstman et al., who reported an implant failure rate of 14.6 % [17]. Implant removal after plate fixation resulted in refracture in 1.0–5.3 % [10, 17–19].

Similar results have been found in the literature regarding minor complications in plate fixation [20], and the incidences of major and minor complications after ESIN seem to comply with estimates from literature elsewhere [21].

Theoretically, complications after plate fixation differ from complications after intramedullary fixation. Plate fixation provides a rigid fixation, originally intended to achieve primary bone healing. Fracture healing occurs without much periosteal ossification, and after fracture healing the plate might still contribute to the mechanical strength of the fixation. Therefore, implant removal might reduce mechanical strength which could explain the slightly increased refracture rates. Another explanation for the tendency of slightly more refractures might be the screw holes after implant removal. These weak spots could potentially initiate a refracture in small clavicles.

Fixation using an intramedullary device results in secondary bone healing. Secondary bone healing is achieved through periosteal ossification, and after fracture healing the intramedullary device does not continue to contribute to the mechanical strength of the fixation. Therefore, intramedullary fixation might show less refractures after removal of the implant.

Implant failures can also be explained by this mechanism. An intramedullary device moves along with the slight movements of the bone and will restore it to its original form. Plate fixation is rigid and does not move. When excessive movement occurs, the plate might bend or break.

The main problem after ESIN is medial protrusion causing irritation or skin perforation resulting in minor revision surgery. In the literature, medial protrusion is reported in the range of 5.2–38.8 % [6, 15, 22, 23]. This minor complication can be prevented by anatomical reduction and fixation of the fracture to prevent telescoping. Early abduction of the arm should also be considered as a cause of medial protrusion of the TEN. Patients should be advised not to abduct the arm over 90° in the first two weeks postoperatively. Another option of reducing medial protrusion is the use of medial end caps. Frigg et al. showed a reduction in medial protrusion rates by using an end cap for TEN [23].

For plate fixation a larger incision is made, which results in a higher risk of infection and probably less cosmetic satisfaction. In this study no significant differences in infection rates between the two groups were observed.

This study is limited by its retrospective design, resulting for instance in an older patient group receiving plate fixation. The impact of the plate fixation group being older could not be assessed in this relatively small patient group. Further, shoulder function and cosmetic appearance were not adequately documented. Therefore, results after both procedures regarding shoulder function and cosmetic appearance could not be compared. However, according to comparative studies on this subject, no significant differences in shoulder function and cosmetic appearance have yet been reported between both techniques [16, 17, 24].

During the study period, different types of plate fixation were used according to fracture type or surgeon’s preference. This might limit the general application of the complications in the plate group. The reason for using different plates was the availability of the types of plates. At the start of this study period reconstruction plates were used, and later the small fragment locking plates became available and were used. However, the main goal of this study was to compare complications after two principles of osteosynthesis for DMCF: plate fixation and intramedullary fixation with ESIN.

Moreover, the retrospective study design may have hampered a complete record of complications that may have occurred during follow-up, but were treated and followed up elsewhere, at another hospital. Due to the surgeon’s preference of treatment method and the retrospective design we also encountered a difference in treatment policy for different types of fractures. Simple fractures (i.e. A2 or B2, Table 2) had a greater possibility of being treated with ESIN than complex fractures.

Currently a randomised controlled study is being performed at the Diakonessenhuis Utrecht comparing plate fixation and intramedullary fixation in DMCF (POP study [25]). The goal of this study is to provide a better insight into results and the complications after both treatments [26]. In this study the frequency of implant failures differed almost significantly for patients treated with plate fixation compared to ESIN. Furthermore, a tendency towards refracture after implant removal and major revision surgery after plate fixation was observed.
